# On the Pathology and Treatment of Chorea

**Published:** 1881-07

**Authors:** Edward C. Mann

**Affiliations:** New York


### ON THE PATHOLOGY AND TREATMENT OF CHOREA.

BY EDWARD C. MANN, MD., OF NEW YORK.

Chorea is a disease of the nervous system, of a convulsive nature, belonging principally to early life, and characterized by irregular and spasmodic movements of the voluntary muscles. These movements take place against the will of the patient, and are usually more marked on one side of the body than the other. They soon become general, however, and are increased by the patient's attempting to exercise his will, or by emotional excitement. The disease generally begins very gradually, and is not noticed for some time. Chorea has a very intimate connection with acute rheumatism and cardiac disease, and many choreic patients will be found to present upon examination an irregular action of the heart, an anæmic murmur at the base of the heart, or evidence of endocarditis, pericarditis, or both. Rheumatism, therefore, and more especially rheumatism complicated with pericarditis, or endocarditis, may be regarded as one of the most prominent causes of chorea. Among other causes that may be mentioned, sudden fright ranks foremost, while anxiety, overwork and ill-health are also predisposing causes.

There is more or less paralysis in chorea, which is indicated by the loss of facial expression, loss of speech, loss of the power of swallowing, dragging of the limbs, inability to hold out the arm without its falling, the readiness with which patients become tired, and the soft, flaccid state of their muscles. Some degree of paralysis is, indeed, quite a marked feature in chorea. A child affected with chorea has a dull, listless expression, avoids associating with other children, does not evince the customary interest in his games and amusements, and becomes incapable of learning his lessons correctly, or recollecting with any degree of accuracy. There is an apparent mental deficiency, and there is more or less emotional disturbance, excessive timidity, capriciousness and fretfulness. The child is restless and fidgety, and ungracefulness of movement becomes very conspicuous. He does not sit long in one place, but is constantly changing his position. He stumbles in going about, or up and down stairs, cannot hold or pass dishes at the table, and generally knocks whatever he holds against something else. The choreic movements usually begin on one side, either of the face, or else one hand and arm are affected. These movements soon involve the whole of one side, and after a few days or weeks, extend to the other side, involving the whole body. If the attack comes on during an attack of rheumatism, no prodromal symptoms may be seen, or if the attack is induced by a sudden emotion the onset is sudden.

The convulsions are very peculiar, and affect to a greater or less extent the whole body, and are of a disorderly, not a rythmical nature. They consist of sudden impulsive movements. They are clonic spasms, which are not stopped until sleep comes. The speech is usually thick and confused, but not lost. When the patient endeavors to answer questions, the convulsive movements of his face and mouth becomes much worse, and he finds it very difficult to articulate. The words come out with a peculiar drawl or stammer. This difficulty of speech may depend upon the respiratory muscles and larynx being affected, as well as upon the convulsive action of the lips and tongue. As a result of the respiratory muscles being involved, the breath is often drawn through the larynx with a suddenness that produces a strange, grunting noise. The convulsive action of the muscles of the head and neck is as irregular as those of the face, so that the head is jerked from one side to the other. The convulsive movements of the upper extremity are more striking than those of the lower. The shoulders are hitched, the arms are moved to and from the side, the forearm is pronated, supinated and flexed, and all sorts of grotesque movements executed. It is very difficult for the patient to hold a glass or cup of liquid to the lips, and it is carried in all directions before it reaches its destination. The legs are affected like the arms, and as soon as the patient tries to use them their action becomes very jerky and uncontrollable. The body is twitched about very violently, into odd and eccentric attitudes. In the most cases, the features, head and neck, are in continual motion. The body is doubled up and writhed about in strange contortions, and the patient's condition is very pitiable to see. The vacant, imbecile aspect of the patient increases as the disease continues, and depends very much upon the involvement of the muscles of expression. Functional or organic disease of the different organs of the body may supervene in the course of an attack of chorea. The leapings and dancings of the religious enthusiasts, as the "jumpers," and the "convulsionnaires," should properly be classed in the category of choreic affections. The people who, in Scotland, were affected with the leaping ague, and with convulsions and dancing fits, also come under this head.

*Prognosis.*—The prognosis is, in the majority of cases, favorable. The general length of time for an attack is from four to six weeks to three or four months. In a small minority of cases the disease lasts for many years or even a lifetime. In a few fatal cases the convulsive paroxysms become aggravated and the spasms are incessant. The patient dies of exhaustion. In the majority of cases the recovery is thorough and complete if the patient is judiciously treated, and the child recovers his mental and physical health, although occasionally the implicated muscles remain feeble and atrophy, or contract.

*Pathology.*—Several hypotheses respecting the morbid anatomy and pathology of chorea have been advanced by different observers, of more or less ability. One, which originated with Dr. Kirkes, and has since been supported by Dr. Hughlings Jackson, adopts the theory of embolism. Dr. Kirkes did not indicate what part of the nervous system he considered to be the seat of the disease, but he said that he considered chorea to "be the result of irritation produced in the nerve centres by fine molecular particles of fibrin, which are set free from an inflamed endocardium, and washed by the blood into the cavities of these centres." Dr. Hughlings Jackson, adopting and enlarging on the theory of Dr. Kirkes, endeavors to show that the emboli are lodged in the vessels of the nerve tissue, forming the convolutions near the corpus striatum—the blood supply of which is derived from the middle cerebral artery—and that a system of under nutrition was induced from a diminished supply of blood.

Dr. Radcliffe accepts Dr. Hughlings Jackson's views so far as clinical evidence can be adduced, and says: "Taking chorea of one side of the body, hemichorea, as the simplest form of chorea, and putting it side by side with hemiplegia, the result of embolism, good reason is found for believing that the disorder of movement and the palsy both point to the region of the corpus striatum as the seat of mischief. If this be the seat of mischief in hemiplegia, why not in hemichorea? The muscles moved in hemichorea are those most palsied in hemiplegia. In hemichorea, as in hemiplegia, the arm, as a rule, is more affected than the leg. In right hemichorea, as in right hemiplegia, the speech is generally very much affected. Again, hemichorea is always more or less mixed up with, and sometimes ends in, hemiplegia; and on the other hand, hemiplegia, from various causes, is not infrequently attended by chorea or movements of some kind or another. The fact that the face is involved in chorea shows that the seat of the disorder must be above the spinal cord. The facts which have been instanced point to the convolutions near the corpus striatum, rather than in any other part of the brain, as the part affected." Dr. Broadhead also accepts the theory of embolism, and considers that embolism of the fine vessels of the sensori-motor ganglia is the principal cause of chorea.

*It does not seem to the writer that the pathological facts which have been elicited by morbid anatomy justify the theory that chorea is produced by or is dependent on inflammatory processes in the brain or cord.* Tremor, convulsion and spasm, do not necessarily depend upon inflammation, but may depend much more readily upon *irritation*, and this irritability may, I think, exist just as well in the thalami optici, corpora quadrigemina, pons varolii, in the medulla or the spinal cord, as in the corpora striata. The appearances in the nervous system, after death, of embolism as the cause of chorea, and the morbid appearance being located in the sensori-motor ganglia, are too few to support this theory successfully, and morbid appearances which are discovered do show that all parts of the nervous system may become affected in the course of chorea. The cord is very often found affected, and particularly the posterior columns, almost enough to suggest a relationship between this disease and locomotor ataxy. Inflammation cannot be essential to chorea, for in some cases there are no traces of inflammation.

The most reasonable theory regarding the production of chorea seems to the writer to be that it primarily proceeds from a morbid irritability of the nervous centres, and that in the subsequent course of the disease any or all parts of the nervous centres *may* become involved in an inflammatory process, but not necessarily so. In many cases there is an inherent irritability of the nervous system, which is easily proved by inquiring into the family history of our patient. Dr. Radcliffe himself avers, that in the more aggravated cases of chorea there is a tendency to run into one or other of the inflammatory diseases of the brain and spinal cord. The general unilateral tendency of chorea, which, as far as it goes, is acknowledged by the writer to point toward diseases of the crus cerebri, corpus striatum or cerebral hemisphere, is offset by the involvement of the muscles of the eyeballs and of the muscles supplied by the upper portions of the facial nerves, which as a rule are not involved in organic lesions of this part. The tendency of chorea to implicate the whole body and the muscles of deglutition and respiration, is also adverse to this hypothesis; and, as I have said, the general resemblance, in many points, of the convulsive movements to those of locomotor ataxy, point to a lesion in the posterior column of the cord. The objection to this would be, however, that if the cord were affected, the disease would not manifest a unilateral tendency. Another very decided objection to the theory of embolism, is the fact of the absence of the disordered movements during sleep. If embolism were present, owing to plugging up of minute cerebral arteries, the lesion would be a constant one, and if this were the cause of the convulsive movements there could be no remission, neither could they abruptly cease, as I have seen them under treatment. Another objection is, that chorea is much more frequent in girls than in boys, while rheumatism, which, by inducing vegetation upon the valves of the heart, is adduced to be the cause, is most frequent in males. Again, the embolic theory entirely fails to explain those cases which are due to fright or anxiety, where the heart is perfectly sound.

The symptoms of chorea are undoubtedly connected with a morbid irritability of the cerebral convolutions, the ganglia at the base of the brain, the pons, the medulla, and the spinal cord. While admitting that chorea is generally associated with rheumatism—in a large proportion of cases with cardiac disease—we maintain that chorea depends upon hyperæmia of the nervous centres, which is produced by rheumatic conditions or by mental or reflex nervous irritability or irritation. There is a general tendency to dilatation of the smaller vessels, and these arterial dilatations are attended with exudation into the tissues immediately surrounding them, and the sclerosis which is thus induced in the tissues surrounding the vessels explains the wasting of the muscles, rigidity of the limbs, and permanent paralysis when it supervenes upon chorea.

*Treatment.*—There are few diseases of the nervous system so difficult to treat successfully as chorea, and few in which so many remedies have been employed. The old treatment of Sydenham, who employed bleeding and tartrate of antimony, has been virtually abandoned, although Sir Thomas Watson recommends local bleeding when there is a fixed pain in the head, and he also uses iron as the favorite medicine in case of chorea, with English practitioners, and Dr. Elliotson especially; forty cases of cure being reported by this mode of treatment.

It has appeared to me that the good accruing from the use of iron is that obtained from improving the general health of the patient, and in this way, as iron is an important tonic, it undoubtedly does good, although I do not think it should be regarded as exerting any specific action in chorea. Sulphate of zinc, in increasing doses, commencing with one grain, three times a day, has been employed, especially in France, where it was introduced as a remedy for chorea by Trousseau, who commenced with doses of gr. 1-28 in children, gradually increasing it until the full physiological effects of the drug were produced, maintaining them for awhile. The iodide and bromide of potassium have also been used, but without practical results. The various narcotics have been tried, but with no good results.

In my own treatment of these cases I endeavor to give the nervous system rest and nutrition. I obtain the former by avoidance of excitement, early hours and the calmative influence of warm baths at bed-time ; the latter by using phosphorated cod-liver oil, or the oil in connection with the phosphide of zinc, 1-16 gr. in pill, three times a day. Gentle gynmnastic exercises are very valuable, and should by no means be neglected. My favorite remedy, and the one which seems to be the nearest to a specific in chorea, is arsenic, which I use hypodermically. To Dr. C. B. Radcliffe, of England, belongs, I think, the credit of being the first to use arsenic in this matter. He first employed it in 1866, and he selected the most tender point over the constructing muscle. In the case he relates, he injected three minims of Fowler's solution, January 12th, in a patient who had suffered for nine years from distressing chorea. On March 21st the patient left the hospital almost well. The injections had reached fourteen minims per day, and marked improvement was noticed from the first. In cases coming to my private retreat for nervous diseases, I used a mixture of equal parts of Fowler's solution and water, to avoid any local irritation which might be produced by the undiluted solution. In children I rarely see any want of toleration of the drug in the system, and rarely, also, in those of older years. I have found that very rapid improvement generally takes place under this treatment from the first, and my patients gain flesh. I generally use electricity in the form specially indicated in individual cases, as an important adjuvant in improving the whole nervous and physical condition of my patients. I commence with three minims of Fowler's solution, and inject, subcutaneously, for a week, every other day, and in the second week increase the dose to five minims every other day, increasing two minims each week, and in from one to two months I obtain a cure. In recent cases a month or six weeks will generally suffice, while in old cases sixty to seventy days may elapse before a cure is accomplished.

In troublesome cases I also use, as an adjuvant, ether spray to the spine, and as I have before indicated, electricity. By the method of using Fowler's solution which I employ, the gastric disturbances which are produced when the medicine is given by the stomach are avoided, and the good results which we can obtain are very much more rapid. I have been very much pleased with the cures I have obtained, and should certainly advise the plan of treatment I have spoken of in chorea, by general practitioners, believing that they will find it, as I have done, most efficacious.—*College and Clinical Record.*

